# Supplementary material for: Evaluation of an evidence-based practice continuing education course for Canadian Naturopathic Doctors
Source: PLoS One. 2025 Jun 3;20(6):e0324452. doi: 10.1371/journal.pone.0324452 (PMC12133167; doi:10.1371/journal.pone.0324452)
Supplement: Supplemental file 1 — (PDF) [file pone.0324452.s001.pdf]

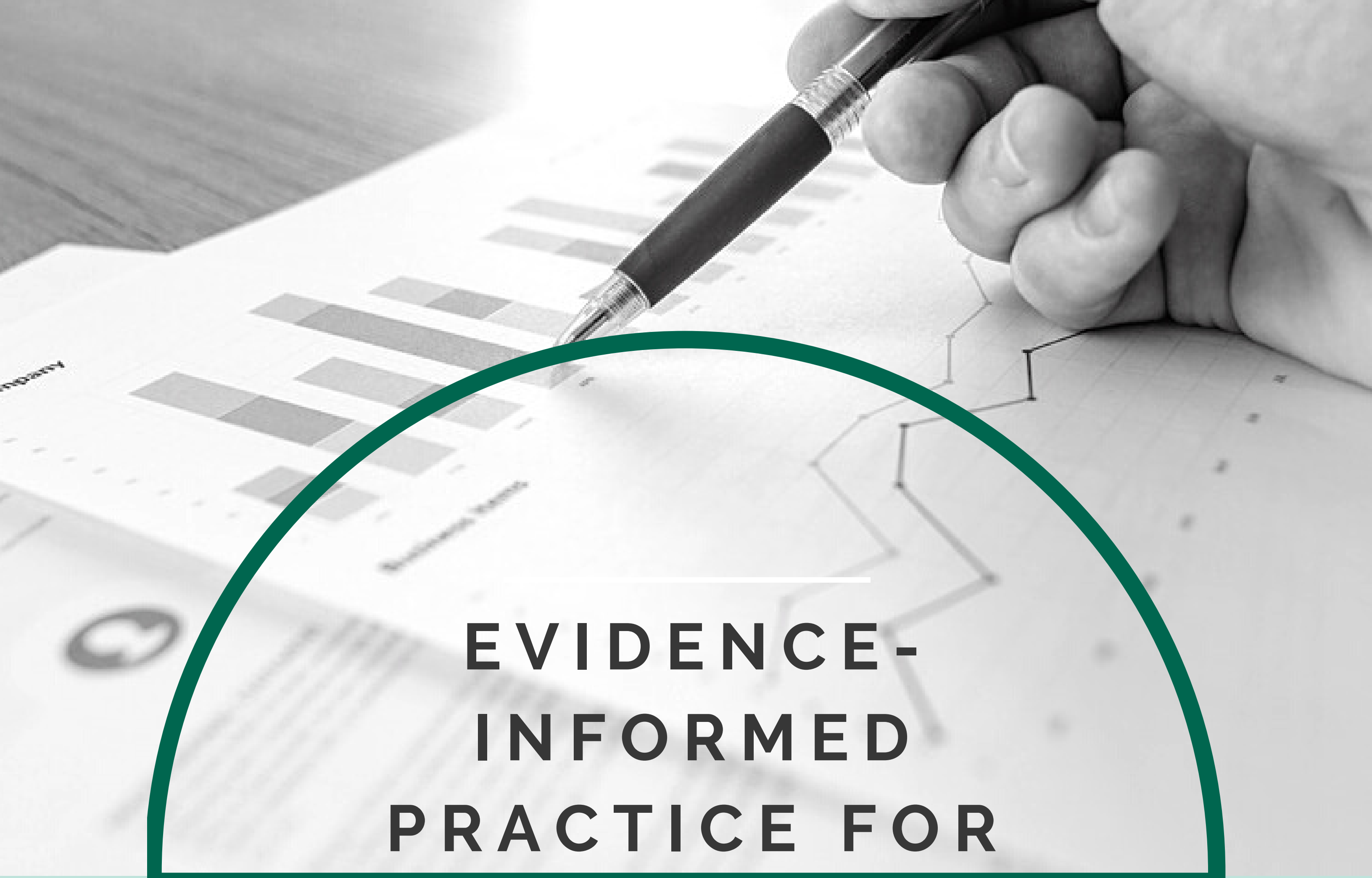

EVIDENCE-INFORMED PRACTICE FOR CANADIAN NATUROPATHIC DOCTORS

CCNM CE COURSE

2021

INSTRUCTOR:
DR. MONIQUE AUCOIN ND
CANADIAN COLLEGE OF NATUROPATHIC MEDICINE

Evidence-Informed Practice

STEPS FOR DOING EIP

**Ask**	Formulate an answerable research question
**Acquire**	Find the best available evidence
**Appraise**	Critically appraise/evaluate the evidence
**Apply**	Apply the evidence by integrating with clinical expertise and patient's values
**Access**	Evaluate performance

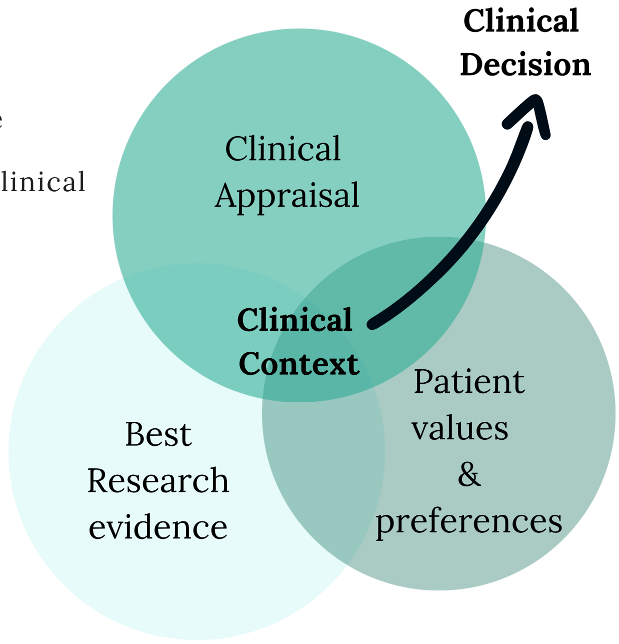

Hierarchy of Evidence

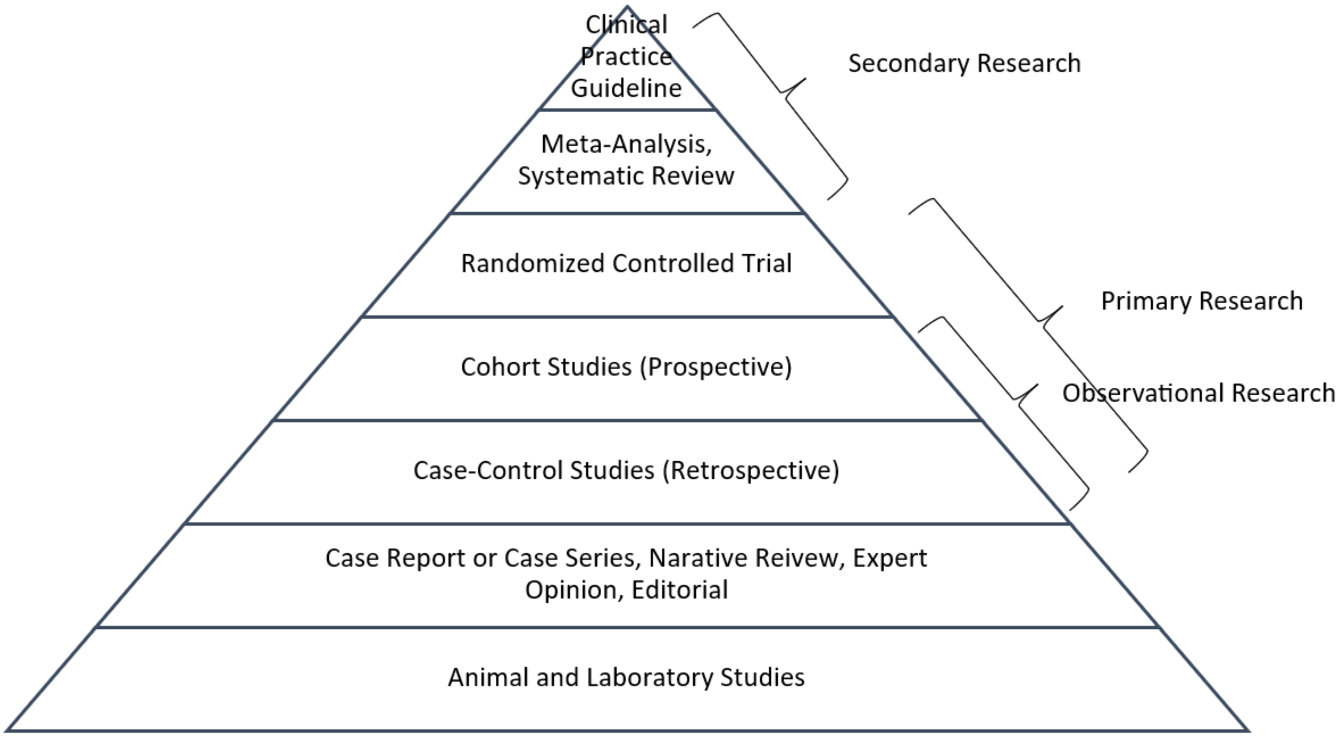

Clinical Trials

FEATURES	USE	STRENGTH	LIMITATIONS
• Intervention is provided<br>• Assess the likelihood of an outcome<br>• Prospective	• Determine cause and effect	• Comparison group accounts for natural progression<br>• Randomization makes the groups balanced at baseline (hopefully!)<br>• Blinding can minimize expectation	• Difficulty (cost, time)<br>• Sample may not be representative<br>• Challenging to study complex/ non-pharm interventions

Consider when critically appraising:

1. Did it involve a clearly defined population (whom do they represent?)
2. What was the method of recruitment?
3. Was allocation truly random?
4. Does Table 1 show baseline demographics and differences between groups?
5. Adequate description of treatment, description of blinding, assessment of compliance?
6. Complete reporting of results, withdrawals (how many and why? included in analysis?)

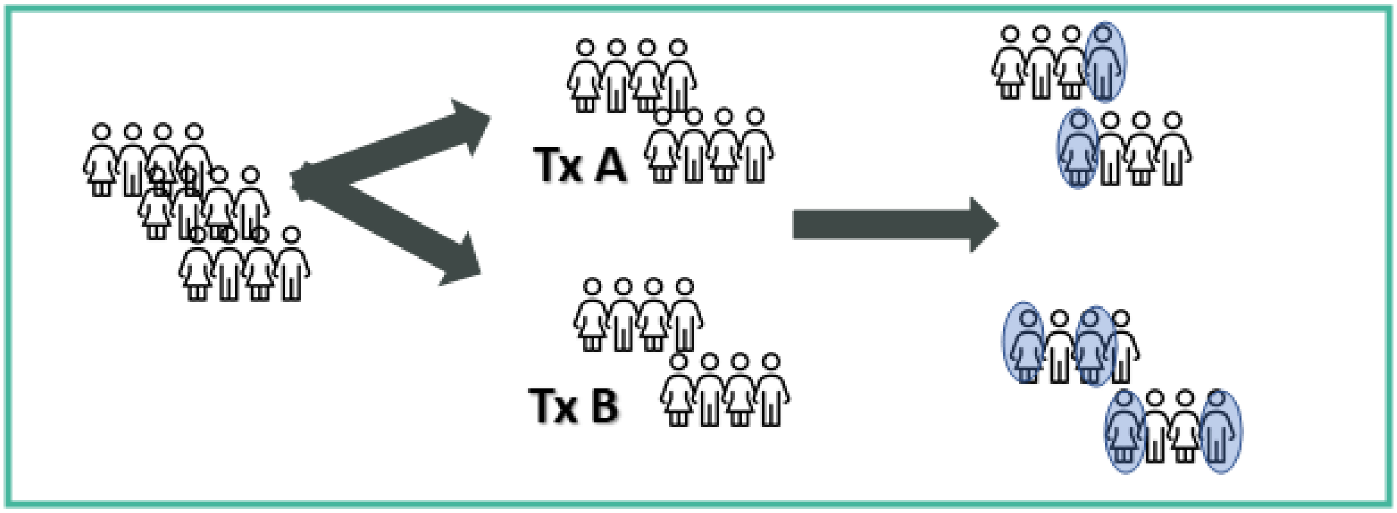

Observational Trials

FEATURES	USE	STRENGTH	LIMITATIONS
• exposure is NOT controlled by the researcher	• is there a relationship between an exposure and an outcome? (correlation not causation)	• can ask any question (even about harm)<br>• case-control/cross-sectional: less expensive, faster, better for rare outcomes<br>• cohort: assess exposure before outcome	• Participants not assigned to groups randomly, may differ<br>• Confounding factors may influence results<br>• Cohort: slower

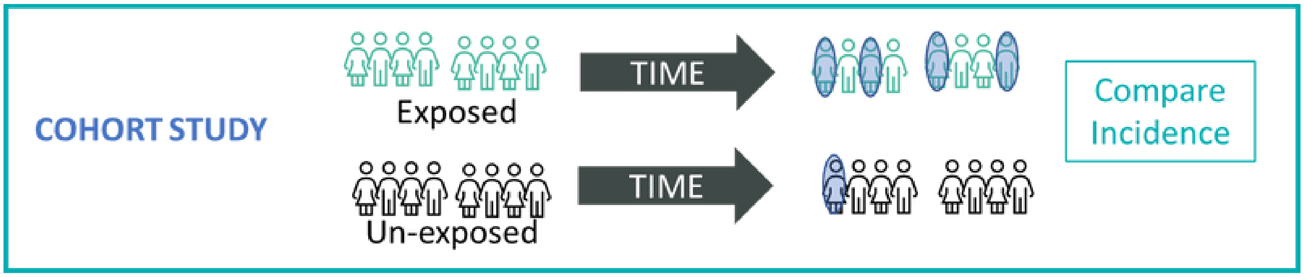

Consider when critically appraising observational studies

1. Do the participants reflect the population?
2. How was the exposure assessed and how was the outcome assessed (reliable and validated methods?)
3. Were confounding factors considered?

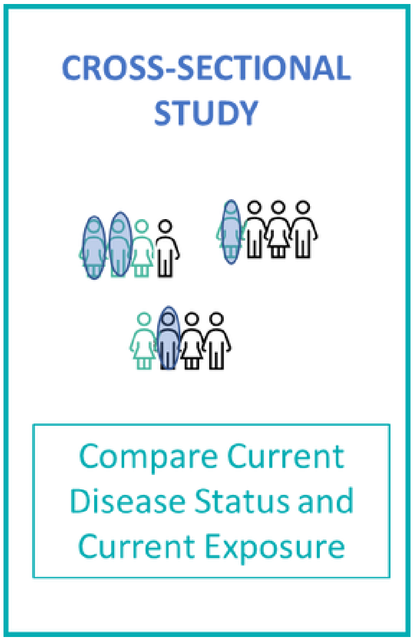

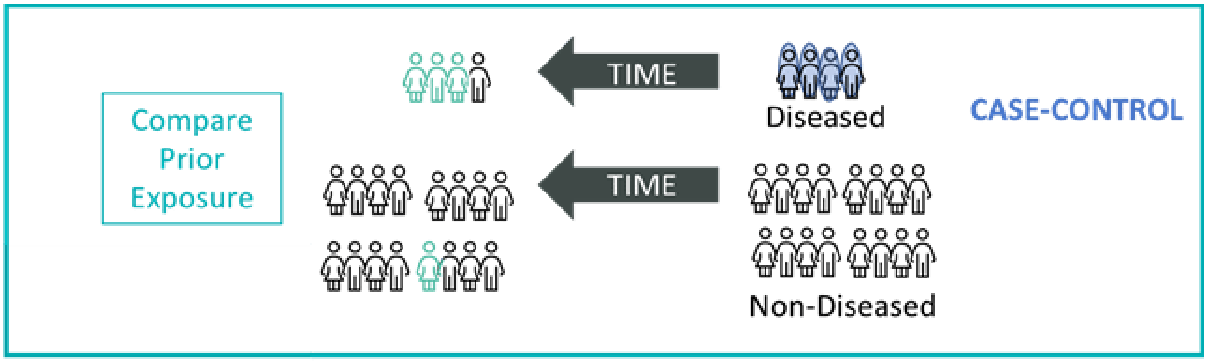

Systemiatic Review and Meta-Analysis

Consider when critically appraising a Systematic Review

1. Clear question (Hint: PICO)
2. Clear inclusion/exclusion criteria for unbiased study selection
3. Comprehensive search strategy (2+ databases)
4. Assessment of individual study quality

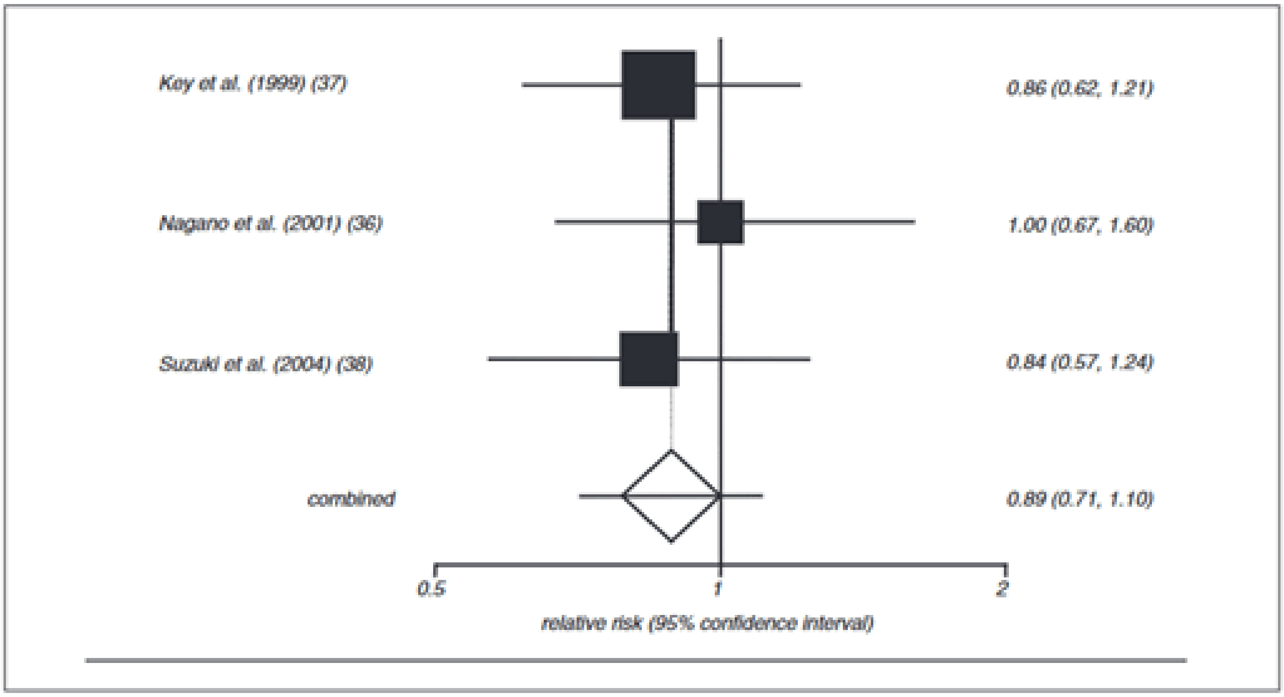

Ref: Seely D et al. The effects of green tea consumption on incidence of breast cancer and recurrence of breast cancer: a systematic review and meta-analysis. Integrative cancer therapies. 2005 Jun;4(2):144-55.

Reading this forest plot: none of the studies reported a significant reduction in risk. The pooled effect is also not significant (confidence interval crosses 1).

Stats Basics for Reading a Paper

TEST FOR STATISTICAL SIGNIFICANCE

P- VALUE	What at the odds that the difference is due to chance; typically p>0.05 (5%) is considered not significant
CONFIDENCE INTERVAL	95% sure the actual value is within this range. If these ranges overlap for 2 values,the 2 values are not significantly different. Ex treatment group: 132.4 (95% CI 120.6-142.9), control group: 139.7 (95% CI 130.7-148.2) -> not significantly different

These depend on: how big is the difference between the groups and how large is the sample size (power)

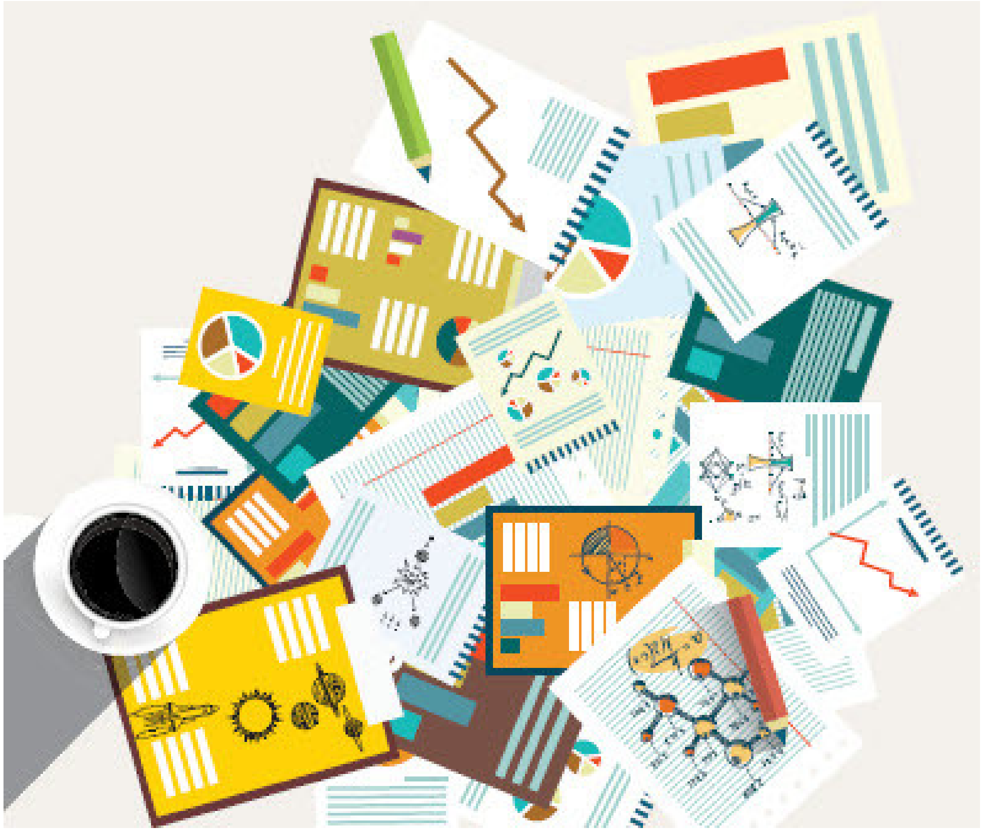

Statistics about Risks

RELATIVE RISK (RR)

Probability of a bad outcome in the intervention group divided by probability of a bad outcome in the control group

$$\text{Relative Risk} = \frac{\text{event rate (tx)}}{\text{event rate (placebo)}}$$

RR = 1 No difference

RR > 1 Risk of bad outcome INCREASED with tx

RR < 1 Risk of bad outcome DECREASED with tx

Ex. Patients taking vitamin C, 12% got URTI, control group, 20% got URTI

RR= 12/20 = 0.6

RELATIVE RISK REDUCTION (RRR)

How much did the treatment reduce the risk of the bad outcome?

RRR= 1-relative risk

- Previous example:
- RR= 12/20 = 0.6
- RRR= 1-0.6 = 0.4 (40% relative reduction in risk with the vitamin C)

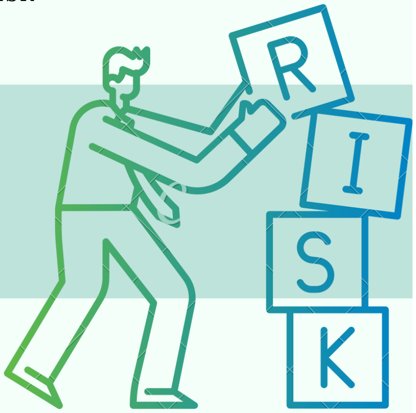

RELATIVE RISK REDUCTION (ARR)

The absolute amount the intervention decreased the risk

ARR= risk of outcome with placebo – risk with mediation

Previous example:
ARR= 20%-12% = 8% (8% relative reduction in risk with vitamin C)

***When outcomes occur infrequently, RRR can be much bigger than ARR

Looking for Scientific Research

P Population
I Intervention
C Comparision
O Outcome

Google Scholar
https://scholar.google.ca/

Great for sourcing free full text (right hand side)

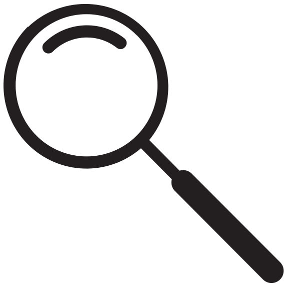

PubMed Tips: https://pubmed.ncbi.nlm.nih.gov/

- Searching Pubmed: https://www.youtube.com/watch?v=aqWuRIzkLo8
- Boolean operators: AND, OR, NOT
  - Ex. (blood pressure OR hypertension) AND (hibiscus OR Hibiscus sabdariffa) NOT tincture
- Truncation * (ex. hyperten* = hypertension, hypertensive...)
- Search Term Subsets:
  - Dietsuppl[sb] -searches a long list of dietary supplements
  - Cam[sb] -complementary medicine topics
  - ex. Hypertension AND Dietsuppl[sb] will search for any dietary supplement studies involving hypertension
- Limits:
  - "Study Type" limit is helpful (**start by looking for a SR/MA, then a clinical trial to quickly identify the strongest evidence)
- MeSH Terms: allow you to clearly specify what you are looking for:
  - Access MeSH database: https://www.ncbi.nlm.nih.gov/mesh/
  - Tutorials: https://www.nlm.nih.gov/mesh/meshhome.html
  - A quick video: https://www.youtube.com/watch?v=ycfCoy6RW3Q

Pre-synthesized Evidence

- Cochrane Library – rigorous SRs and MAs www.cochrane.org
- Natural Medicines – evidence summaries for interventions and medical conditions, interaction checker; access through CAND membership website ND portal;
- Trip Databases -displays findings based on the evidence hierarchy www.tripdatabase.com

Sourcing Full-Text Articles

- Google Scholar
- Research Gate www.researchgate.net
- Email the corresponding author

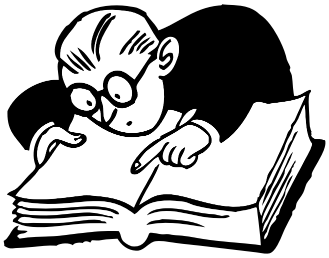

Critical Appraisal

Many Tools to Choose From!

- CASP checklists https://casp-uk.net/casp-tools-checklists/ **This is the one we practiced in class
- Centre for Evidence-Based Medicine Critical Appraisal Tools https://www.cebm.ox.ac.uk/resources/ebm-tools/critical-appraisal-tools
- BMJ Best Practice Critical Appraisal Checklists https://bestpractice.bmj.com/info/toolkit/ebm-toolbox/critical-appraisal-checklists/
- Cochrane Risk of Bias – more intensive, often used for systematic reviews https://methods.cochrane.org/bias/resources/rob-2-revised-cochrane-risk-bias-tool-randomized-trials
- Free training available: https://training.cochrane.org/rob-2-learning-live-webinar-series
- There are also many tools for the quality of reporting available at the EQUATOR Network https://www.equator-network.org/

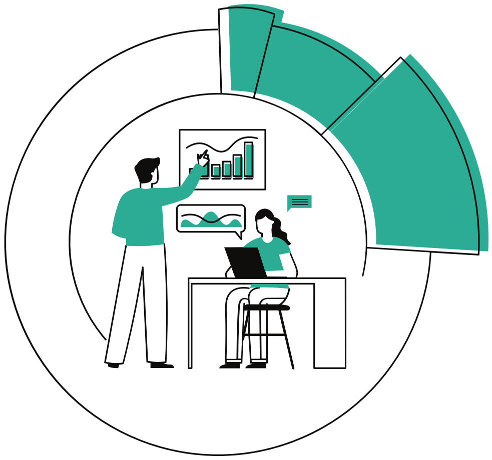

Handling Dropouts

- Per-protocol analysis: analyze people who completed the intervention (exclude dropout); may overestimate the effect of the intervention
- Intention to treat analysis: analyze people based on the group they were assigned to, whether or not they complete the study (include dropouts); cautious, may underestimate the effect
- Helpful video: https://www.youtube.com/watch?v=Kps3VzbykFQ

Applying Evidence to Clinical Practice

Outcomes

- Clinical outcome: a clinical event, ex. stroke
- Surrogate outcome: an indicator that can be observed sooner, at a lower cost or less invasively, ex. LDL cholesterol

Shared Decision Making

- Quick read: https://www.ahrq.gov/health-literacy/professional-training/shared-decision/tool/resource-2.html
- More detail: Coulter and Collins 2011

Applying Research to Naturopathic Medicine:

BEST EVIDENCE	CLIICAL EXPERTISE	PATIENT PREFERENCE	PHILOSOPHY/ PRINCIPLES

Interpret and apply evidence in the context of the principles
Scientific evidence may not tell us if it is consistent with the philosophy, this involves reflection by a clinician (ex. Does this therapy address the root cause of illness?)

What if the research evidence is contradictory?
Step 1: Look for a systematic review
Step 2: SQR approach to prioritize studies

Decision Aid tools: condition-specific tools to assist patients in making a decision

- https://carethatfits.org/tools/
- https://decisionaid.ohri.ca/index.html

**Strength**	Think about the hierarchy of evidence; focus on the highest-ranking studies
**Quality**	look for differences in quality (randomization, blinding, analysis)
**Relavence**	How relevant was the study to the patient (characteristics/preferences), practitioner (scope/expertise), intervention (access/cost), context (setting)

Getting Involved in Naturopathic Research

WRITING A CASE REPORT

Purpose: report something that is not well documented in the existing literature (ex. a success, a failure, an adverse event, a rare manifestation, a new disease, a unique course of illness)

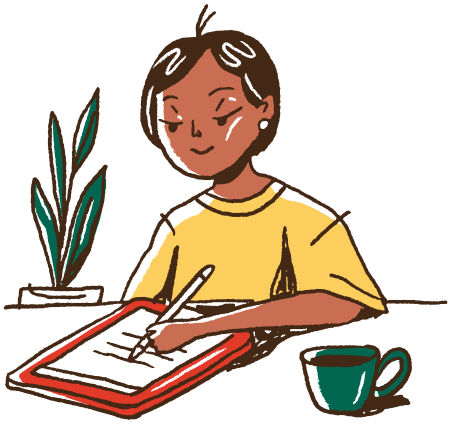

CARE Guidelines for writing case reports: https://www.care-statement.org/

TITLE	Include the words "case report"
INTRODUCTION	Background on the condition and intervention<br>What is the gap in the literature that you are addressing?
PATIENT INFO	Primary concern, medical/fam/psychosocial history
CLINICAL FINDINGS	Physical exam findings
DIAGNOSTIC ASSESSMENT	Lab test, imaging findings
THERAPEUTIC INTERVENTIONS	Type of intervention, dose, changes in the intervention
FOLLOW UP & OUTCOMES	Clinician- and patient-assessed outcomes, follow-up diagnostic and other tests, adherence and tolerability, adverse events
DISCUSSION	Strengths and limitations of your report<br>Situate within existing medical literature (how does it add)<br>Take away/conclusions (may include needs for further research)
OTHER	Can include the patient perspective<br>Informed consent should be obtained
