# Supplementary material for: Evaluation of an evidence-based practice continuing education course for Canadian Naturopathic Doctors
Source: PLoS One. 2025 Jun 3;20(6):e0324452. doi: 10.1371/journal.pone.0324452 (PMC12133167; doi:10.1371/journal.pone.0324452)
Supplement: Attachment — Submitted filename: PLOSONE.docx [file pone.0324452.s002.docx]

**Title:** Evaluation of an Evidence-Based Practice Continuing Education Course for Canadian Naturopathic Doctors
**Article type:** Research Article
**Authors:** Monique Aucoin; Genevieve Newton; Matthew Leach; Kieran Cooley

Feedback:

The abstract is well-structured and aligns with the general expectations for publication in *PLOS ONE*, but there are a few areas that could be strengthened to enhance its suitability. Find attached an evaluation based on *PLOS ONE*'s criteria:

**Strengths:**

1. **Clear Background & Rationale:**
   - The introduction effectively establishes the importance of Evidence-Based Practice (EBP) for Canadian Naturopathic Doctors (NDs) and justifies the need for evaluating a Continuing Education (CE) course.
2. **Methodological Clarity:**
   - The pre-post study design is clearly described.
   - The use of validated tools (Evidence-Based Practice Attitudes and Utilization Survey) and objective knowledge assessment adds credibility.
   - The follow-up assessment at 2 months strengthens the study’s rigor.
3. **Results & Interpretation:**
   - The study reports quantitative outcomes (percentage of satisfaction, significance of knowledge and skill improvement).
   - The discussion acknowledges the mixed feedback and provides a balanced interpretation.
4. **Conclusions & Future Directions:**
   - The findings are summarized effectively, and the abstract suggests improvements for future iterations of the course.

**Areas for Improvement:**

1. **Clarity on Statistical Analysis & Significance:**
   - The abstract mentions a "significant increase" in knowledge and skill but does not specify the statistical tests used or provide p-values.
   - While *PLOS ONE* does not require significance testing, clarity on effect size or confidence intervals would enhance transparency.
2. **Addressing Limitations:**
   - The abstract does not mention limitations (e.g., small sample size, self-selection bias, short follow-up duration).
   - Stating potential biases would align with *PLOS ONE*’s commitment to transparency.
3. **Better Framing of Null Findings (Attitudes & Use of Evidence):**
   - The lack of change in attitudes and evidence use is mentioned but not explored.
   - A brief interpretation (e.g., time constraints, need for longer interventions) could add depth.
4. **Terminology & Readability:**
   - The phrase “Preliminary evidence suggests...” could be more precise.
   - Instead of “Preliminary evidence,” consider “Findings suggest...” or “Results indicate...” since data was collected and analyzed.

**Suitability for *PLOS ONE*:**

✅ *PLOS ONE* values methodological rigor, clear research questions, and data transparency, all of which are present in this abstract.
✅ The study has a clear real-world application and contributes to healthcare education, aligning with *PLOS ONE*'s broad scope.
❌ Minor improvements are needed in statistical reporting, discussion of null results, and acknowledgment of limitations.

**Verdict:**

**Likely suitable for publication in *PLOS ONE*, but revisions would strengthen the submission.**
