# Supplementary material for: Evaluation of an evidence-based practice continuing education course for Canadian Naturopathic Doctors
Source: PLoS One. 2025 Jun 3;20(6):e0324452. doi: 10.1371/journal.pone.0324452 (PMC12133167; doi:10.1371/journal.pone.0324452)
Supplement: Attachment — Submitted filename: Reviewer report.docx [file pone.0324452.s003.docx]

**PONE-D-25-02800 "Evaluation of an Evidence-Based Practice Continuing Education Course for Canadian Naturopathic Doctors".**

Comments to the Authors

Thank you for your work on this manuscript. This research will add to the literature in a number of different areas. The manuscript is well-written and meets the journal’s publication criteria.

Originality

This is an original piece of work that can add to the literature in the context of designing courses that focus on evidence-based practice (EBP) in the context of continuing education for naturopathic doctors.

Introduction

Relevant background information and a rationale for the study are provided. Study aims are clearly stated.

Methods

The Methods section is clearly organised and provides relevant information under each section. Study measures are clearly described. Detailed information of the analysis process is provided.

Results

The results are clearly outlined and answer the research aims. The Tables and Figure support the results.

Discussion

The main findings of the study are discussed, and some links are made to earlier research that is applicable. Study limitations and strengths are discussed. Conclusions are supported by the data. Future research directions are discussed.

**Suggested edit**

1. Instead of using the term ‘complementary and alternative medicine,’ consider using the term ‘traditional, complementary, and integrative medicine (TCIM).’
